# Merkel cell carcinoma in a 38-year-old man: a case report^⋆^

**DOI:** 10.1016/j.abd.2022.05.009

**Published:** 2023-06-29

**Authors:** Yixin Liu, Wenjing Liang, Qiongrong Chen, Yongchang Wei

**Affiliations:** a Department of Radiation and Medical Oncology, Zhongnan Hospital of Wuhan University, Wuhan, China; b Department of Pathology, Zhongnan Hospital of Wuhan University, Wuhan, China

Dear Editor,

Merkel Cell Carcinoma (MCC) is a rare but aggressive cutaneous cancer, and it occurs mostly in older Caucasians, especially in immunocompromised patients. It is reported that up to 80% of MCC is associated with Merkel Cell Polyomavirus (MCPyV) infection, and 20% is related to ultraviolet.^1^ The clinical presentation of MCC is nonspecific and varied, but most commonly presents with rapidly growing, solitary violaceous nodules with or without ulceration. Approximately 26%‒36% of MCC patients have lymph node involvement and 6%‒16% present with distant metastasis at their initial visit.^2^ Surgery and radiotherapy are first-line treatments, while an emerging effective treatment modality is Immune Checkpoint Inhibitor (ICI).^3^

Herein, we report an extremely rare case of MCC in a young man with rapid deterioration to provide experience for the diagnosis and treatment.

## Case report

**Figure 1:**
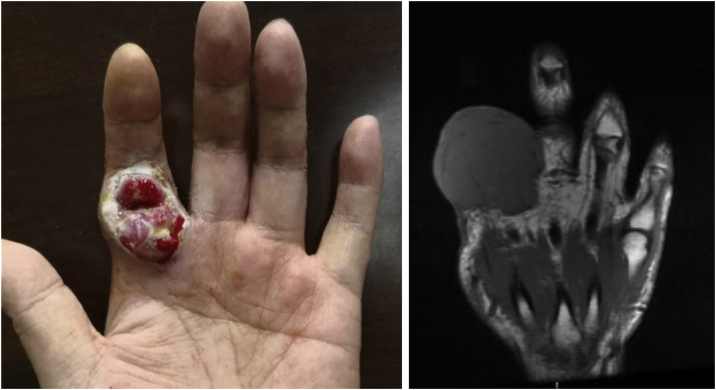
Clinical images of skin lesion, July 2020. (A) Red-purple painless nodule in the left index finger. (B) A representative MRI image

A 38-year-old man with a five-year history of unspecified lesion on the left index finger presented to our clinic for an asymptomatic nodule at the same site. In the beginning, he presented with an eczematous lesion on the left index finger in 2015 and just accepted ointment treatment. Then an erythematous nodule appeared and was removed by surgery in 2016 without pathological examination. A growing red-purple painless nodule measuring 52×51×39 mm appeared at the same site four years later, which seemed to be covered with small, widened vessels (Fig. 1). Moreover, physical examination showed dark red nodules with scaly scabs on his right thenar and right ankle. And he reported no trauma to these lesions and no systemic symptoms such as fever and weight loss. He was a dentist, lived in urban areas without an epidemic and denied family medical history and long-term administration of drugs. Moreover, HIV infection was ruled out.

**Figure 2:**
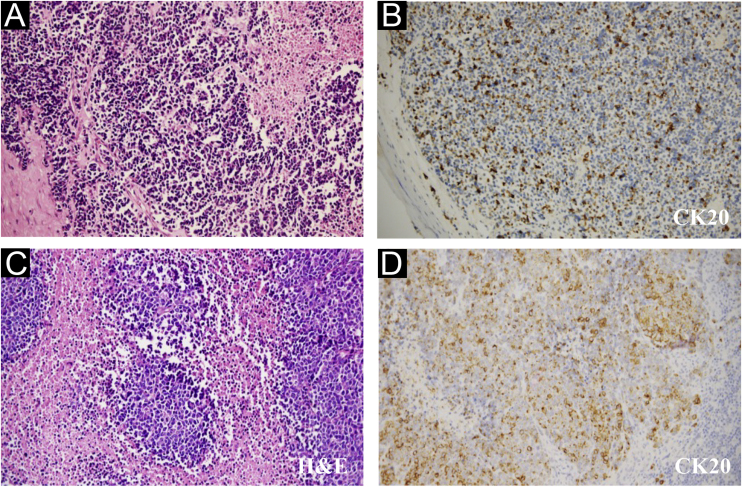
Pathological features of the finger neoplasm (A‒B) and left axilla lymph nodes (C‒D). (CK20, Cytokeratin 20, 200×)

**Figure 3:**
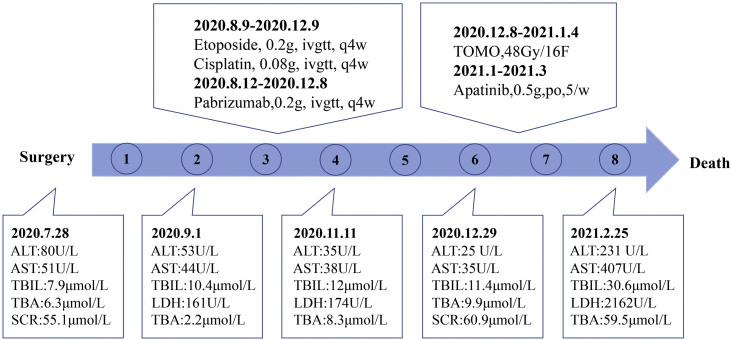
Time Course. Blue dots indicated passing months after surgery

**Figure 4:**
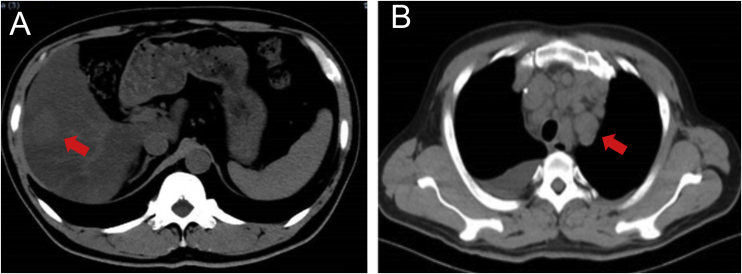
Representative images of the patient. (A) Liver involvement, December 2020. (B) Multiple lymphadenectasis, February 2021

Wide excision of left index finger neoplasm and axillary lymph node dissection was performed in July 2020. The results of Hematoxylin-eosin staining and immunohistochemistry were in line with MCC histopathologic characteristics (Fig. 2). Besides, the pathological diagnosis for the other two lesions was Squamous Cell Carcinoma (SCC) and surgery was performed. The patient then accepted adjuvant therapy (Etoposide, Cisplatin, Pembrolizumab), routine blood tests, and renal and liver function were carefully monitored (Fig. 3). The disease maintained stable for four months. However, the liver was invaded by MCC in December 2020 (Fig. 4A), which suggested the adjuvant treatment was no longer effective. Given that he was still young, we offered a local radiotherapy (48Gy/16F) for hepatic metastases and tyrosine kinase inhibitor Apatinib was administered with the patient’s consent. In February 2021, he presented with severe clinical worsening, and multiple enlarged cervical lymph nodes were observed (Fig. 4B). The patient eventually died 7 days after this admission.

The present report refers to an MCC patient under the age of 40 with poor therapeutic effect. Owing to its rarity, almost MCC treatment recommendations are derived from retrospective studies, and we take several potential factors that account for his poor immunotherapy response.

The first one to consider is age. Paulson^4^ reported a higher fraction of metastases and a more aggressive course in younger MCC patients. MCC frequently was misdiagnosed due to non-specific clinical characterization and the patient mentioned above was treated as eczema for five years, which prevented him from timely treatment. Besides, there was a study indicating that neuroendocrine carcinomas associated with SCC had a higher incidence of local recurrence.^5^ In our report, it is also noteworthy that the patient repeatedly appears lesions in the same skin site. But it is difficult to identify their connection because no pathological examination was carried out before this admission.

The Next Generation Sequencing results of peripheral blood showed no somatic variation within tumor genomes. What’s more, this patient had a low tumor mutation burden (TMB) score (bTMB-L <1 Muts/Mb) as well as relatively lower PD-L1 expression level (5%‒10%) and CD8+ Tumor-Infiltrating Lymphocytes (TILs) density (10.01 psc/mm^2^), which are all immune-response related indicators in MCC.^6^ In addition, MCC located in the extremities and involving the liver is also an important adverse feature.^7^, ^8^

The patient in this case had higher malignancy and poorer therapeutic response owing to multiple factors. Therefore, we emphasize the importance of early biopsy, as well as adequate evaluation before treatment.

## Financial support

None declared.

## Authors’ contributions

Yixin Liu: Data collection, writing of the manuscript.

Wenjing Liang: Data collection, critical review of the manuscript.

Qiongrong Chen: Critical review of the literature.

Yongchang Wei: Approval of the final version of the manuscript.

## Conflicts of interest

None declared.
